# One R or the other – an experimental bioethics approach to 3R dilemmas in animal research

**DOI:** 10.1007/s11019-024-10221-y

**Published:** 2024-08-17

**Authors:** Christian Rodriguez Perez, David M. Shaw, Brian D. Earp, Bernice S. Elger, Kirsten Persson

**Affiliations:** 1 Institute for Biomedical Ethics, University of Basel, Basel, Switzerland; 2 Care and Public Health, Research Institute Maastricht University, Maastricht, Netherlands; 3 Oxford Uehiro Centre for Practical Ethics, University of Oxford, Oxford, UK; 4 Centre for Biomedical Ethics, National University of Singapore, Singapore, Singapore; 5 Center of Legal Medicine, Faculty of Medicine, University of Geneva, Geneva, Switzerland; 6 Institute for Animal Hygiene, Animal Welfare and Farm Animal Behaviour, University of Veterinary Medicine Hannover, Hannover, Germany

**Keywords:** Experimental bioethics, Animal research, 3Rs principle, 3R dilemmas, Researchers’ decision-making

## Abstract

Sacrificial dilemmas such as the trolley problem play an important role in experimental philosophy (x-phi). But it is increasingly argued that, since we are not likely to encounter runaway trolleys in our daily life, the usefulness of such thought experiments for understanding moral judgments in more ecologically valid contexts may be limited. However, similar sacrificial dilemmas are experienced in real life by animal research decision makers. As part of their job, they must make decisions about the suffering, and often the death, of many non-human animals. For this reason, a context-specific investigation of so-called “3R dilemmas” (i.e., dilemmas where there is a conflict between the principles of replacement, reduction, and refinement of the use of animals in research) is essential to improve the situation of both non-human animals and human stakeholders. An approach well suited for such investigation is experimental philosophical bioethics (“bioxphi”), which draws on methods similar to x-phi to probe more realistic, practical scenarios with an eye to informing normative debates and ethical policy. In this article, we argue for a need to investigate 3R dilemmas among professional decision-makers using the tools of bioxphi. In a first step, we define 3R dilemmas and discuss previous investigations of professionals’ attitudes in such cases. In a second step, we show how bioxphi is a promising method to investigate the *whys* and *hows* of professional decision-making in 3R dilemmas. In a last step, we provide a bioxphi template for 3R dilemmas, give recommendations on its use, explore the normative relevance of data collected by such means, and discuss important limitations.

## Introduction

Sacrificial dilemmas such as the trolley problem played an important role historically in ethics and more recently in experimental ethics, as part of experimental philosophy (x-phi) (Greene 2023). However, it is increasingly argued that, since we are not likely to encounter runaway trolleys in our daily life, the usefulness of such thought experiments to understand moral decisions in more ecologically valid contexts may be limited (Bauman et al. 2014). Thus, a perceived need for more realistic, practical, and well-contextualized insights has played a role in driving the so-called “empirical turn” in (bio)ethics (Borry et al. 2005). However, stark sacrificial dilemmas are in fact routinely experienced in real-life by professional animal research decision-makers (e.g., researchers, members of animal research ethics committees, veterinarians, animal welfare specialists, authorities). As part of their job, they must make decisions about the suffering, and often (causing) the death, of dozens, hundreds or even thousands of non-human animals (hereafter referred to as animals). Such sacrificial dilemmas arise, for example, in cases of 3R dilemmas (i.e., dilemmas where there is a conflict between the principles of replacement, reduction and refinement of the use of animals in research).

Animal research is a debated social issue. Opponents question the need for such research and highlight the harm to animals. Supporters argue that animal research benefits the public, which also funds much of this research through taxes. Either way, the public is a stakeholder in this area. In a democracy, the public has the power to shape the future of animal research (e.g., by voting on regulations, as Swiss citizens did in 2022 regarding an initiative aimed at banning animal experimentation; see Bradley 2022), and public attitudes surveys are thus increasingly conducted in this area (Ipsos 2018; Whittaker et al. 2022). But when it comes to decisions on concrete cases (e.g., sacrificial dilemmas), the public is almost never involved. While patient and public involvement in such ethical decision-making does exists (e.g., through participation in committees), it is still the exception (for the European context, see Olsson et al. 2016). To address this, there are now calls for more direct inclusion of representatives of the public, especially patients who might stand to benefit from animal research (Davies et al. 2024).

Nevertheless, in keeping with most current regulations, decisions about sacrificial dilemmas in animal research are still undertaken by professionals, such as researchers or members of ethics committees with a technical background. Thus, although we endorse recommendations to investigate public attitudes on this issue (and are engaged in such investigations ourselves), in this work, we outline a research agenda for understanding the decisions of professionals faced with sacrificial animal dilemmas. Plausibly, knowing more about the factors and processes shaping decision-making in these dilemmas can help inform both theoretical and policy-oriented discussions around key ethical issues (e.g., compassion fatigue and moral distress among professionals, guidance in 3R dilemmas, minimization of the harm inflicted on research animals).

To achieve this aim, we argue it is necessary to go beyond simply cataloguing *what* option(s) these decision-makers may choose when confronted with such scenarios to understanding *why* or *how* they do so. This is where the methods of experimental philosophical bioethics—sometimes shortened to experimental bioethics or simply “bioxphi” (a portmanteau of bioethics and x-phi)—come into play. Like its parent discipline of x-phi, bioxphi uses experimental methods drawn from the cognitive sciences to understand the factors and processes that shape relevant aspects of human cognition; but like its other parent discipline, empirical bioethics, it seeks to do so in a way that largely eschews abstract or unrealistic scenarios in favor of more ecologically valid phenomena of interest to bioethicists, all with an eye to informing substantive normative debates and/or on-the-ground ethical policymaking (Lewis et al. 2023). However, in contrast to many studies within empirical bioethics, which tend to employ standard questionnaires to document *what* various stakeholders think about certain issues, bioxphi uses controlled experiments to investigate the *whys* and *hows* of appropriately contextualized moral decision-making (Earp et al. 2021). Thus, it aims to ensure that results are clear and interpretable while still striving for ecological validity (Mihailov et al. 2021).

In this article, we argue for a need to investigate 3R dilemmas among professional decision-makers using the tools of bioxphi. In a first step, we define 3R dilemmas and discuss previous investigations of professionals’ attitudes in such cases. In a second step, we show how bioxphi is a promising method to investigate the *whys* and *hows* of professional decision-making in 3R dilemmas. In a last step, we provide a bioxphi template for 3R dilemmas, give recommendations on its use, explore the normative relevance of data collected by such means, and discuss important limitations.

## 3R dilemmas and professionals’ decision-making

### What are 3R dilemmas?

*3R* refers to the principle of replacement, reduction and refinement of animal use in research, and is now a widely-accepted ethical framework for such research worldwide. First introduced in 1959 by British scientists W. M. S. Russel and R. L. Burch, it is now part of standard guidelines and regulations in many countries. While definitions of the terms *replacement*,* reduction and refinement* might differ from one institution to another (Tannenbaum and Bennett 2015), the purpose of 3R by almost all accounts is to minimize the harm inflicted on research animals, by replacing them with non-animal alternatives, reducing the number of animals used, and refining the designs (i.a., procedures, husbandry) in accordance with animals’ welfare interests. Some jurisdictions have also clarified that the ultimate goal of 3R is the complete replacement of animal research (e.g., the European Union with its Directive 2010/63/EU).

**Fig. 1:**
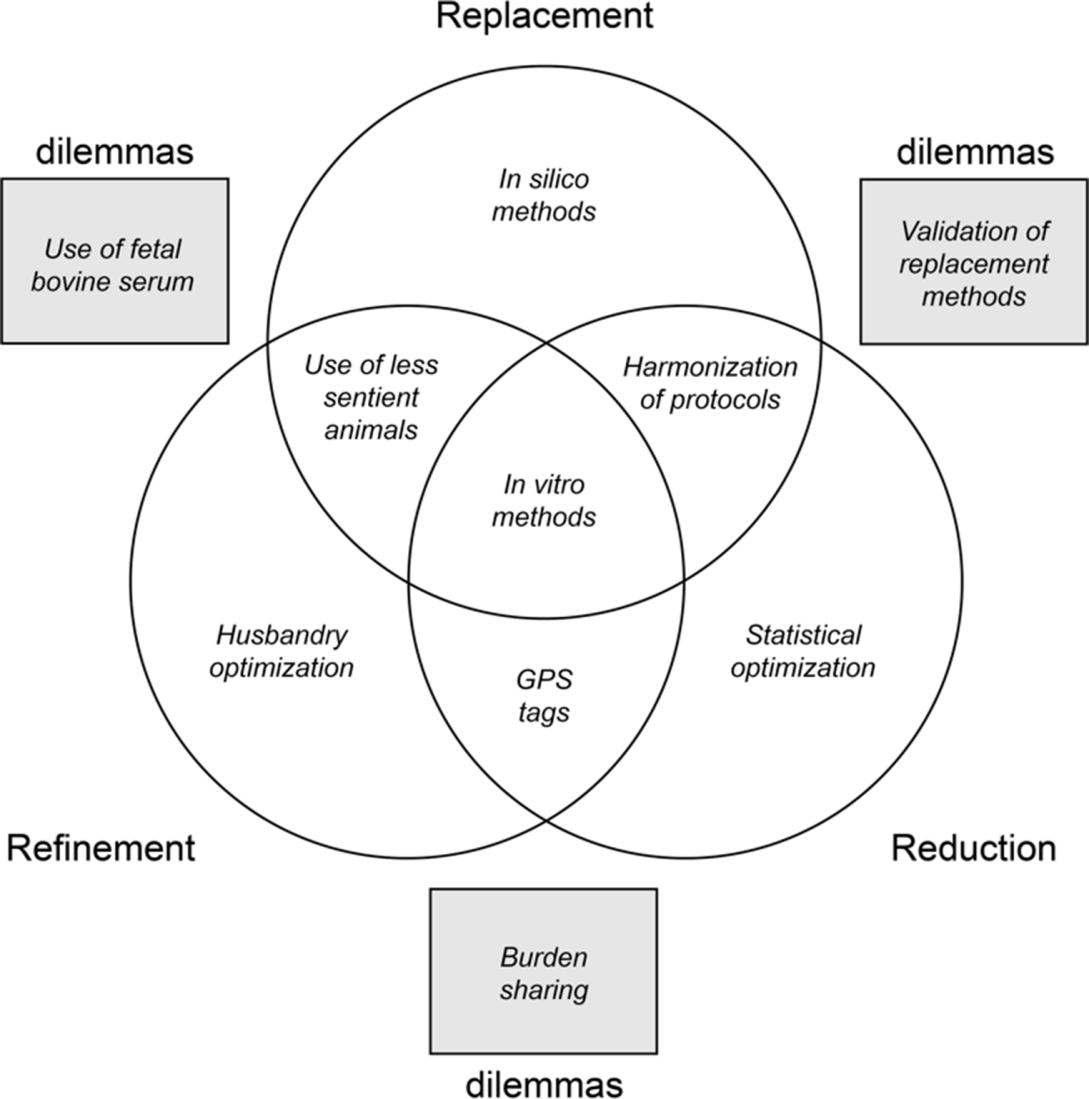
3R interaction with single examples

As is the case for the four “canonical” principles of biomedical ethics (i.e., respect for persons/autonomy, nonmaleficence, beneficence, and justice; see Baker 2024 for a critical discussion), principles of 3R can come into conflict. When such conflicts are not easily resolved (i.e., when it is not clear which option is the least harmful or most protective of animal welfare), we talk about 3R dilemmas. Figure 1 below illustrates the interaction between the Rs by giving one example per category. As we see, an animal research decision might contribute to one R (e.g., statistical optimization), two Rs (e.g., harmonization of protocols) or even all of the three Rs (e.g., use of in vitro methods). For example, by harmonizing protocols, research team B can use data from research team A to answer their research question, thus avoiding the need for animal research. In such a case, they will not only have replaced animals with a non-animal alternative (i.e., data from team A), but also reduced the number of animals used (i.e., animals have only been used in team A’s research). In the case of 3R dilemmas, decisions contribute to one R while being in violation of another R.

An example of a replacement-reduction dilemma is the validation of replacement methods. To validate non-animal alternative methods, it is often necessary to test their efficiency in comparison to animal-based methods, a process which implies the use of animals. This creates a 3R dilemma, because choosing to validate the replacement method would contribute to replacement while being in violation of reduction. When it comes to replacement-refinement dilemmas, one example is the use of fetal bovine serum in replacement methods. Fetal bovine serum is used as a serum-supplement in some non-animal alternative methods (e.g., in vitro cell cultures), but collecting it involves harm to the fetus (Jochems et al. 2002). This also creates a 3R dilemma, because choosing to use fetal bovine serum would contribute to replacement while being in violation of refinement. Finally, refinement-reduction dilemmas can be illustrated by cases of burden sharing. In such cases, a given number of procedures must be performed in a way that allows the procedures to be divided between several animals. This creates a 3R dilemma, because inflicting less harm on individual animals by increasing the number of animals would contribute to refinement while being in violation of reduction, and reducing the number of animals by inflicting more harm on individual animals would contribute to reduction while being in violation of refinement. It is precisely this last type of dilemma that we will focus on in the next sections.

While researchers might unexpectedly face such dilemmas during the conduct of the research (e.g., unexpected circumstances forcing a choice between inflicting more harm to the animals or request additional animals), most 3R dilemmas should be dealt with while designing projects. Indeed, since most states require some sort of ethical approval to use animals in research, decisions about 3R dilemmas must be made, and often justified, when submitting the research design to an animal research ethics committee (AREC). But the fact that 3R dilemmas exist in theory does not mean that decision-makers experience them as such in practice. Decision-makers might, for example, consider only one option and still judge that they applied 3R. Moreover, even if they do consider competing options, their decision could be based on technical, economic, legal, or logistical considerations (e.g., prioritizing reduction over refinement due to the costs of involving more animals). But such considerations should nonetheless be part of the ethical discussion. For these reasons, as we will emphasize in the next sections, it is important to understand the decision-making context and process in animal research.

Nonetheless, if the purpose of 3R is to minimize the harm inflicted on research animals, then in 3R dilemmas the option(s) resulting in the smallest amount of harm should be chosen. In this sense, there are right and wrong options in 3R dilemmas (i.e., setting aside true or essentially unresolvable dilemmas in which a comparable amount of harm, or an equivalent wrong, is guaranteed no matter which option one chooses from a given set). Of course, that is not to say that we have the means to know the right option, or even to choose it confidently in most cases. One reason for this is that harm evaluation is challenging both in relation to live animal welfare (e.g., properly conceptualizing or measuring it) and to complex types of harm such as death. A second challenge is the scope of the evaluation, because inflicting more harm in a specific experiment or research could be the right option in the long term. That could be true, for example, if replacement is prioritized in the replacement-reduction dilemma presented above. In such cases, more animals could be used to develop a non-animal alternative method which will replace so many animals in the future that it will, in the end, be the option causing the smallest amount of harm. Finally, since all things are never really equal in such dilemmas, one could argue that additional harms should enter the equation, such as that experienced by decision-makers in phenomena such as compassion fatigue and moral distress (Grimm 2023; King and Zohny 2022, Reynolds 2018; Randall et al. 2021).

The challenges to our ability to choose the right option confidently in 3R dilemmas should not, however, cause us to abandon the goal of optimizing our decision-making. Quite the opposite, in fact: it is the pursuit of this goal through interdisciplinary scientific progress, debate and collaboration which allows us to make better and more well-thought-out decisions. Therefore, we can trust that on many occasions decision-makers do indeed choose well-justified options in 3R dilemmas, often with the support of experts (i.a., animal welfare specialists, statisticians). In the same spirit, if we want to continuously optimize our decision-making in 3R dilemmas, one thing is clear: the more we learn about the underlying factors of the decision-making process, the more we can improve the situation of both animals and professionals.

### Professionals’ decision-making in 3R dilemmas: previous investigations

Empirical research in animal ethics involving stakeholders can be useful for a variety of purposes, such as identifying moral issues that have escaped the attention of ethicists, analyzing moral opinions and reasoning patterns of stakeholders, or making ethics more context-sensitive (Persson 2015; de Vries and Gordijn 2009). When it comes to previous investigations of professionals’ attitudes to 3R dilemmas, the work of Franco and colleagues (Franco and Olsson 2014, Franco et al. 2018) is foundational. In this section, we consider two of their studies to illustrate these attitudes and highlight, in a next step, how the bioxphi approach can help such investigations.

In their 2014 publication titled “Scientists and the 3Rs: attitudes to animal use in biomedical research and the effect of mandatory training in laboratory animal science”, Franco and Olsson presented refinement-reduction dilemmas to scientists based in Portugal. In particular, the authors aimed at testing the impact of a laboratory animal science (LAS) course on scientists’ attitudes to those cases. The first case related to the housing of mice, as we can see in Table 1 below. As the authors write, most respondents prioritized refinement (i.e., the approach that implied less harm to individual animals, even when that would require using twice as many animals), both before (87%) and after (91%) the LAS course (Franco and Olsson 2014). This methodology was focused exclusively on *which* option is chosen. Although the authors were interested in whether taking a LAS course might influence this choice, they did not employ random assignment to condition (e.g., LAS course versus control course, or no course). Thus, although the percentage of participants choosing each option appears to be slightly different before versus after the LAS course, it is not possible to determine precisely what, if anything, about the course may have causally contributed to these changes.

**Table 1:**
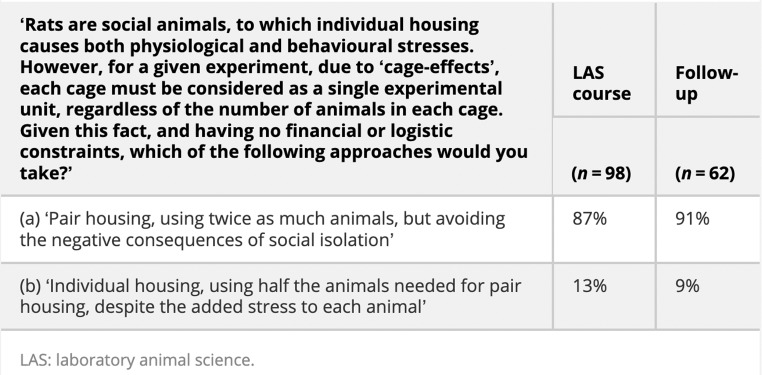
Franco and Olsson (2014) - case study 1

The second case was a burden sharing dilemma, as we can see in Table 2 below. In this case, after prioritizing either reduction (i.e., (a) ’20 trials on the same animal - one per day for 20 days’) or refinement ((b) ’20 trials distributed among 20 animals’) with respect to mice, respondents were presented with a follow up question asking if they would use the same approach if the given species was dogs, rhesus monkeys, chimpanzees, or rabbits (Franco and Olsson 2014).

In this sense, the collected data addressed not only *which* option is chosen by respondents, but also *how*, if at all, the species of animal involved affects their decision. As we will argue in the next section, the bioxphi approach, with its use of controlled experiments involving random assignment to different conditions, is ideally tailored to investigate the influence of such factors. Even so, the results of Franco and Olsson reveal key insights, albeit based on a standard questionnaire (i.e., species were not systematically manipulated in a controlled experiment, but rather were presented in the same manner to all participants in a ‘within-subjects’ design, as sequential options to consider). As the authors write, “researchers were more divided” regarding the prioritization of refinement or reduction in this burden sharing dilemma, compared to the previous dilemma about mice housing (Franco and Olsson 2014). Moreover, it appears that when it comes to certain species, researchers are less willing to use more animals, even if that implies inflicting more harm on individual animals. Indeed, while the vast majority of researchers who prioritized reduction in respect to mice stated that they would use the same approach for the other species, around a third of researchers who prioritized refinement in respect to mice stated that they would change their approach (i.e., prioritize reduction instead) if the given species was dogs, rhesus monkeys, or chimpanzees.

**Table 2:**
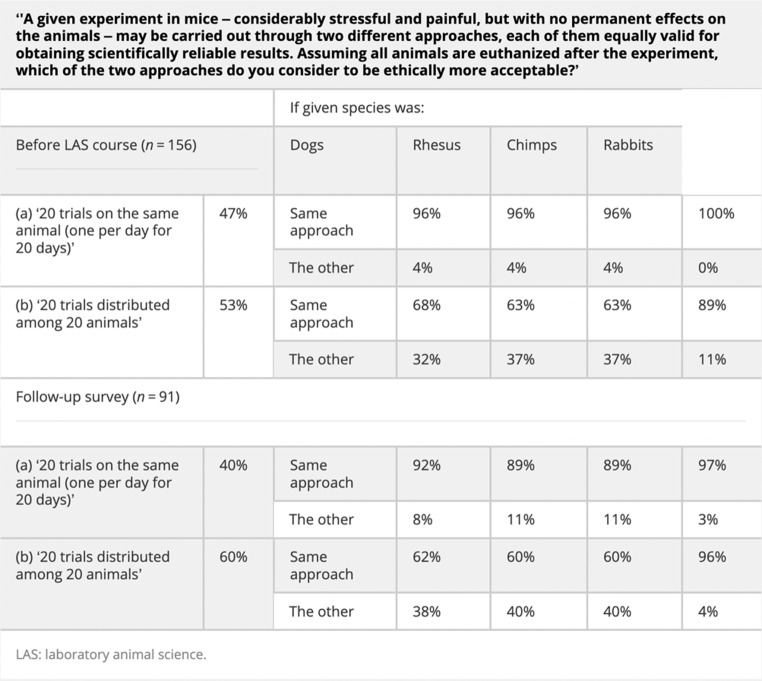
Franco and Olsson (2014) - case study 2

**Fig. 2:**
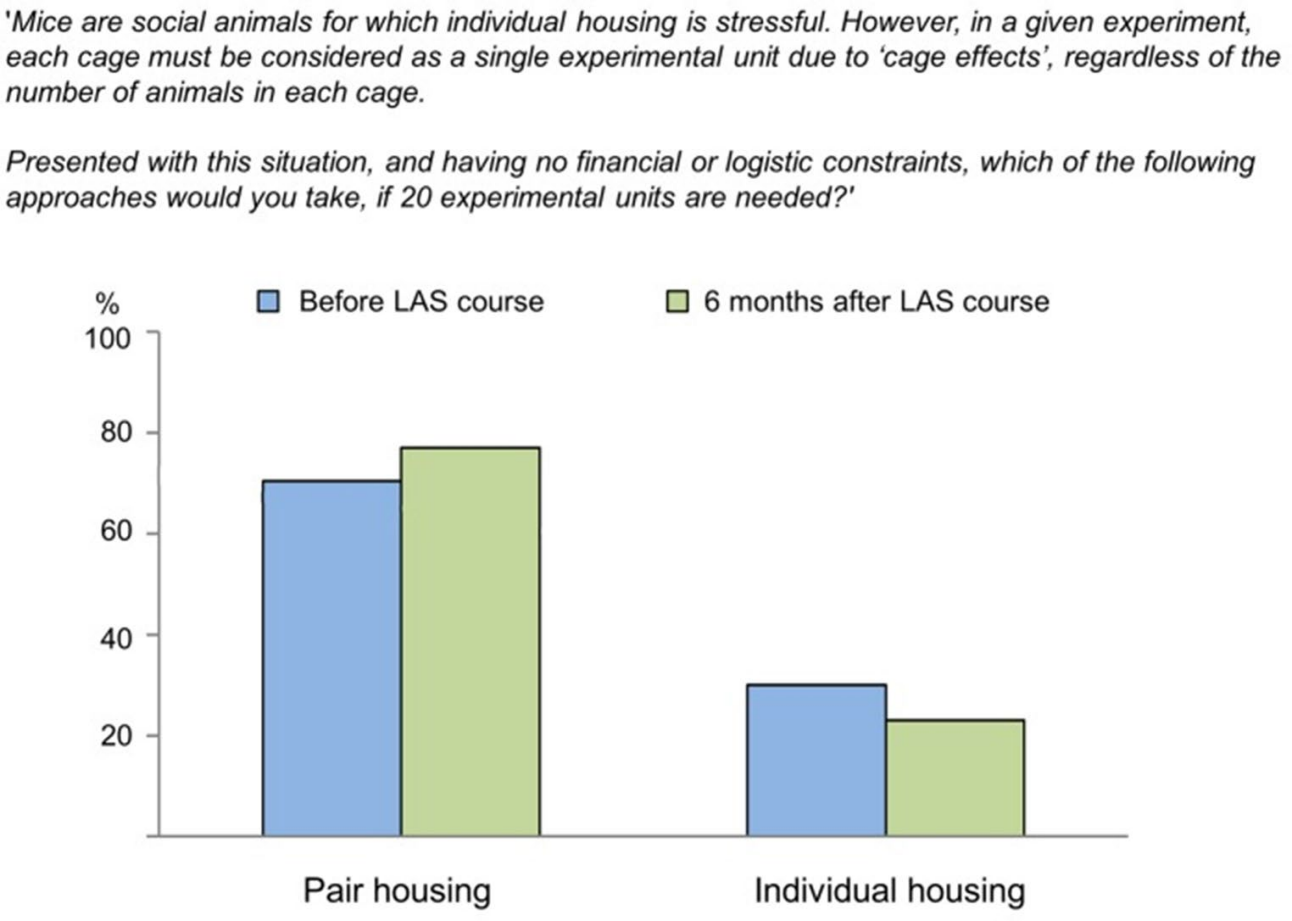
Franco et al. (2018) housing dilemma

More recently, as part of their 2018 publication titled “Researchers’ attitudes to the 3Rs - An upturned hierarchy?” (Franco et al. 2018), Franco, Sandøe, and Olsson presented new results about the refinement-reduction dilemma relating to the housing of mice. As Fig. 2 below shows, results were consistent with Franco and Olsson’s (2014) previous study on this particular case. This time, respondents (i.e., 233 researchers before taking a LAS course and 97 researchers after taking the course) were based in Portugal, Germany, Switzerland, and Denmark. Here again, the method was focused on *which* option is chosen, without probing factors that potentially influence the decision.

Franco and colleagues’ empirical investigations of professionals’ attitudes to 3R dilemmas, and more specifically to refinement-reduction dilemmas, lead the way for further research. Their results not only show that researchers can be divided depending on the type of dilemma (i.e., housing or burden sharing), but also that probing factors such as the species of the animal can reveal key insights. In the next section, we will show why the bioxphi approach is a promising method to investigate the *whys* and *hows* of professional decision-making in 3R dilemmas.

## The need for bioxphi in 3R dilemmas

### Why bioxphi?

As an interdisciplinary “offspring” of both experimental philosophy (x-phi) and empirical bioethics, bioxphi aims not only at establishing *what* judgments or decisions are made by various stakeholders in ethically-charged situations—such as 3R dilemmas—but also at revealing *why* or *how* they judge or decide as they do, by probing relevant situational factors and psychological mechanisms (Earp et al. 2020). To better explain this, the analogy to Marr’s levels of computation might fruitfully be used (Alexander et al. 2010). Marr defines three levels of analysis of a computation in an information processing system, which can be compared to human decision-making as Table 3 below shows.

**Table 3:** Marr’s three levels of analysis compared to human decision-making

Marr’s levels	Human decision-making
1. Computation	Decision - *what*
2. Algorithm	Underlying factors and processes – *why / how*
3. Implementation	Physical implementation – *biology*

In the case of 3R dilemmas, questions at level 1 (Decision - *what)* would be: *What percentage of researchers prioritize refinement*,* reduction or neither in scenario X?* and *How sure are they about their decision?* To answer such questions, a standard questionnaire or input-output kind of study is sufficient.

Questions at level 2 (Underlying factors and processes - *why / how)* would be: *How does the killing of animals at the end of the experiment influence the prioritization of refinement or reduction in scenario X?* or *How does the order of the cases presented affect the prioritization of one of the Rs?* To answer such questions, a suitable approach is to probe factors and processes in controlled experiments.

A question at level 3 (Physical implementation – *biology*) would be: *Which regions of the brain (e.g.*,* linked to emotions*,* memory*,* logical thinking) activate when big numbers are involved and what are the implications?* For this kind of question, adequate technology such as fMRI would be needed.

While questions at all levels would benefit from the context-specificity of empirical bioethics, questions at level 2 and 3 specifically require probing situational factors and psychological mechanisms in controlled experiments. By combining x-phi’s controlled experiments with empirical bioethics’ context-specificity, bioxphi can provide answers to questions at level 2 and, to the extent that the appropriate technology is available, at level 3. It is in this sense that the bioxphi approach goes beyond previous investigations of professionals’ decision-making in 3R dilemmas, enabling deeper exploration of the relevant factors and processes. For this reason, we argue that bioxphi is a key and core method to investigate the *whys* and *hows* of 3R dilemmas decision-making. While investigation at level 3 would also contribute to important findings in 3R dilemmas, in the remaining of the article we will mainly focus on questions at level 2 (*whys* and *hows*).

One key aspect which differentiates bioxphi from x-phi is its emphasis on ecological validity to ensure that results are as generalizable as possible within the specific context under study. To conclude our case for the use of bioxphi in 3R dilemmas, we will now discuss ecological validity in the context of 3R dilemmas.

### Ecological validity in 3R dilemmas

One of the main causes of the so-called “empirical turn” in ethics is the need for context-specific insights. When we consider cases of emergency sacrificial dilemmas like the ones experienced in ER (emergency rooms), it seems clear that investigating them using contrastive vignette techniques (CVT) (i.e., vignettes that describe a particular situation but differ by a specific detail that is expected to impact on participant responses; see Reiner 2019), will most likely miss relevant situational factors (e.g., social relationships, physical environment) and psychological mechanisms (e.g., fear or distress) of real-life cases. Striving for ecological validity in such cases (i.e., the ability to generalize experimental findings to real-life settings) thus requires creative thinking about how best to experimentally capture the relevant factors and processes that likely influence ER decisions. For that reason, the field has given attention to the possible use of immersive technologies to recreate high-fidelity situational experiments, such as VR (virtual reality) (Mihailov et al. 2021).

As we have seen, however, most researchers’ decisions about 3R dilemmas must be made, and justified, when submitting a research design for ethical approval. That means that such decisions are not emergency-type sacrificial dilemmas decisions like the ER case presented above, but rather the result of a process which takes time, and among other aspects involves deliberation with peers, literature review as well as financial and deadline considerations. Recreating exact real-life experiences of 3R dilemmas is therefore complex, and immersive technologies are not of much use here. Nonetheless, one tool provides an opportunity to optimize ecological validity: the application to perform an animal experiment (APAE).

Indeed, in countries such as Switzerland^1^ and the United Kingdom^2^, submissions of animal research designs to ARECs are made through an APAE, in which aspects related to 3R must be explained and justified. The European Union, Canada and the United States also tend to favor such applications, at a national, regional, or institutional level.^3^ In these countries, we can therefore be certain that research teams facing 3R dilemmas must fill out and submit an APAE, either in physical (e.g., written documents) or digital (e.g., digital documents, online platform) form. From the initial application to final approval, the process is known to be demanding. Its duration depends on the country’s regulations, the research team’s completion speed, the frequency of AREC’s meetings and their workload, as well as potential requests to review the APAE. While qualitative research is needed to further investigate the precise role APAEs play in animal research design, everything indicates that this mandatory and crucial step for approval is decisive for 3R dilemma decision-making. For all these reasons, we can be confident that, by analyzing the APAE procedure of a specific country, we will find relevant aspects to probe in controlled experiments. Key aspects of 3R dilemmas, such as the number of animals used, their species, their fate at the end of the experiment or the severity of the experiment, are indeed addressed in most countries’ APAEs. We call these aspects key because, normatively speaking, they are relevant to the aim of minimizing the harm inflicted on research animals. Therefore, we argue that these aspects must be probed with the tools of bioxphi, using the APAE’s specificities of the context under investigation to favor ecological validity.

**Fig. 3:**
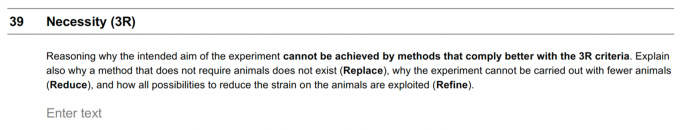
Justification of 3R in Swiss government’s APAE – PDF document

There are indeed relevant differences to be found in comparing different countries’ APAEs on these key aspects. Let’s compare, for example, 3R justification in the APAEs of Switzerland and the UK. As Fig. 3 below shows, the Swiss government’s APAE provides a single textbox for applicants to justify why “the experiment cannot be achieved by methods that comply better with the 3R criteria”. Moreover, it links 3R with the term “Necessity” and provides short definitions of replacement (i.e., a method that does not require animals does not exist), reduction (i.e., the experiment cannot be carried out with fewer animals) and refinement (i.e., all possibilities to reduce the strain on the animals are exploited), which must be used to explain their decisions. It is interesting to notice that no section of the Swiss APAE explicitly asks applicants to address 3R dilemmas. The best we can find is the use of the comparative in the sentence “comply better with the 3R criteria”, which implies that an optimal design, according to 3R criteria, must be chosen.

**Fig. 4:**
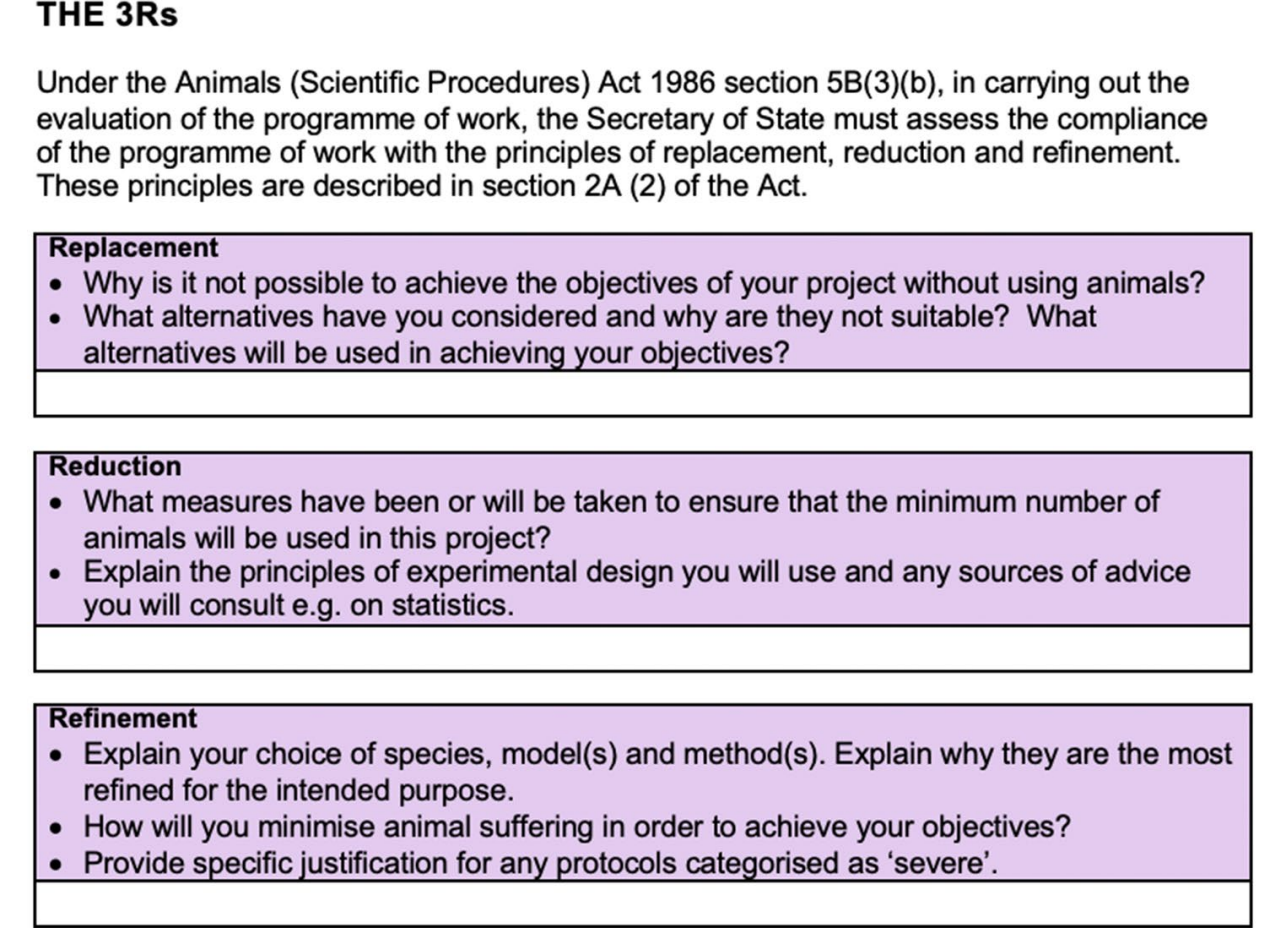
Justification of 3R in UK’s APAE – Microsoft Word document

As we can see in Fig. 4 below, the UK’s APAE does not provide definitions of the Rs, but rather mentions the Act under which they are described. There is not only one textbox in this case, but rather three (i.e., one per R) for applicants to explain how 3R has been considered in their design decision. Additionally, explanations are required about specific aspects of replacement (e.g., What alternatives have you considered and why are they not suitable? ), reduction (e.g., Explain the principles of experimental design you will use and any sources of advice you will consult e.g. on statistics) and refinement (e.g., Explain your choice of species, model(s) and method(s)). Unlike in the Swiss case, there is no mention of an “optimal design” according to 3R criteria, in addition to the lack of a section dedicated to 3R dilemmas.

As we see, while 3R justification must be addressed in both cases, there are relevant differences to be found. Such differences can also be found for other key aspects of 3R dilemmas, and it is necessary for our bioxphi approach to consider them. In the next sections, we will focus on the Swiss APAE to provide a bioxphi template for 3R dilemmas.

## A bioxphi template for 3R dilemmas

### Swiss version for refinement-reduction dilemma

All animal experiments in Switzerland are subject to authorization. While applicants must submit the APAE to their respective cantonal commission for animal experiments, the form which must be filled is the same for all applications. The APAE can be filled either using a Microsoft Word document called “Form A” or directly in a web application called “animex-ch”. Therefore, in real-life, all Swiss-based researchers wanting to perform an animal experiment would need to fill this APAE with their team.

By analyzing the Swiss APAE, we find at least 7 key factors for 3R dilemmas that we consider here, as Table 4 below shows.

**Table 4:** 3R dilemmas key factors in the Swiss APAE

Factor	Description	Section in APAE
Species	Specification of the animals’ species and potential genetic modifications	9
Numbers	Listing of animals used with numbers and groups	9
Origin	Specification of where the animals come from	9
Procedures	Description of the procedures and manipulations on the animals	25
Fate	Description of the method of euthanasia or of the use of the animal at the end of the experiment	27 & 37
Severity	Definition of the maximum expected degree of severity (stress) for each animal group	36
Justification	Provide the reasoning of why the experiment cannot comply better with the 3R	39

As we said, these factors are key because, normatively speaking, they are relevant to the aim of minimizing the harm inflicted on research animals. Indeed, while species, numbers, and severity (which is linked to the procedures) are often thought to be the main measures to assess an experiment’s direct harm, origin and fate can be considered as indirect harms. In the case of origin, this is because bringing animals into existence, breeding them in (approved or non-approved, Swiss or foreign) facilities, transporting them or acquiring them from previous experiments, all have normative implication and potentially involve harm. Regarding fate, the relevance is that killing the animals, using anesthetics or concussion techniques, reusing them in an additional experiment or rehoming them in an animal sanctuary, also are decisions involving ethical principles and values, and might involve harm as well. Finally, 3R justification is also a key normative factor in 3R dilemmas, because getting it right or wrong could determine whether unnecessary harm will be caused.

Secondary factors, which are also present in the Swiss APAE, might also play a role in 3R dilemma decision-making and could be used in the template. For example, duration of the project, purpose of the experiment, husbandry conditions or weighing of interests. The bioxphi approach does not imply that one can gather definitive answers on a phenomenon by experimentally testing a finite number of parameters. In fact, investigating key factors might only be a first step, but will already provide important findings.

As an example, let us consider the investigation of the fate factor (i.e., euthanasia, rehoming, reuse) more closely. Under Swiss law, killing research animals (e.g., as a procedure to collect tissue) with compliant methods is considered as a degree of severity 0 (FSVO 2022).^4^ Experiments with a degree of severity 0 do not require an evaluation by an AREC to be approved, as they are not considered harmful (AniPO, art. 139, 4). In this sense, death (in itself) is not considered as a harm to the animals, nor as worthy of an ethical evaluation, under Swiss law. This is true independently of the number of animals requested. Nonetheless, initiatives for the rehoming of laboratory animals have emerged within the Swiss scientific community in recent years, supporting the idea that giving them “a life after the experiment” is desirable (EPFL 2022). While there are several barriers to rehoming (i.a., it only makes sense for laboratory animals that are still in good health after the experiment and only if their new home can ensure their welfare (Palmer et al. 2023), Swiss law does not allow the rehoming of genetically modified animals), it is seen as beneficial for several reasons (Skidmore 2024). Among these reasons is the view that rehoming improves the life of laboratory animals and the fact that it boosts staff morale (Skidmore 2024). This relates to the impact of killing animals on animal research professionals, reflected by the above-mentioned phenomena of compassion fatigue and moral distress, and suggests that death (in itself) matters (at least to researchers). Valuing their lives in this way, while at the same time dealing with a law considering their death as almost ethically irrelevant, can be considered as a tension. In this context, insights about the weight given by professionals to the continued existence of research animals (i.e., rehoming) and to their death (i.e., euthanasia) in different conditions of 3R dilemmas is important to inform normative debates and policymaking. In the next section we will discuss further how such normative inferences can be made from the results.

**Fig. 5:**
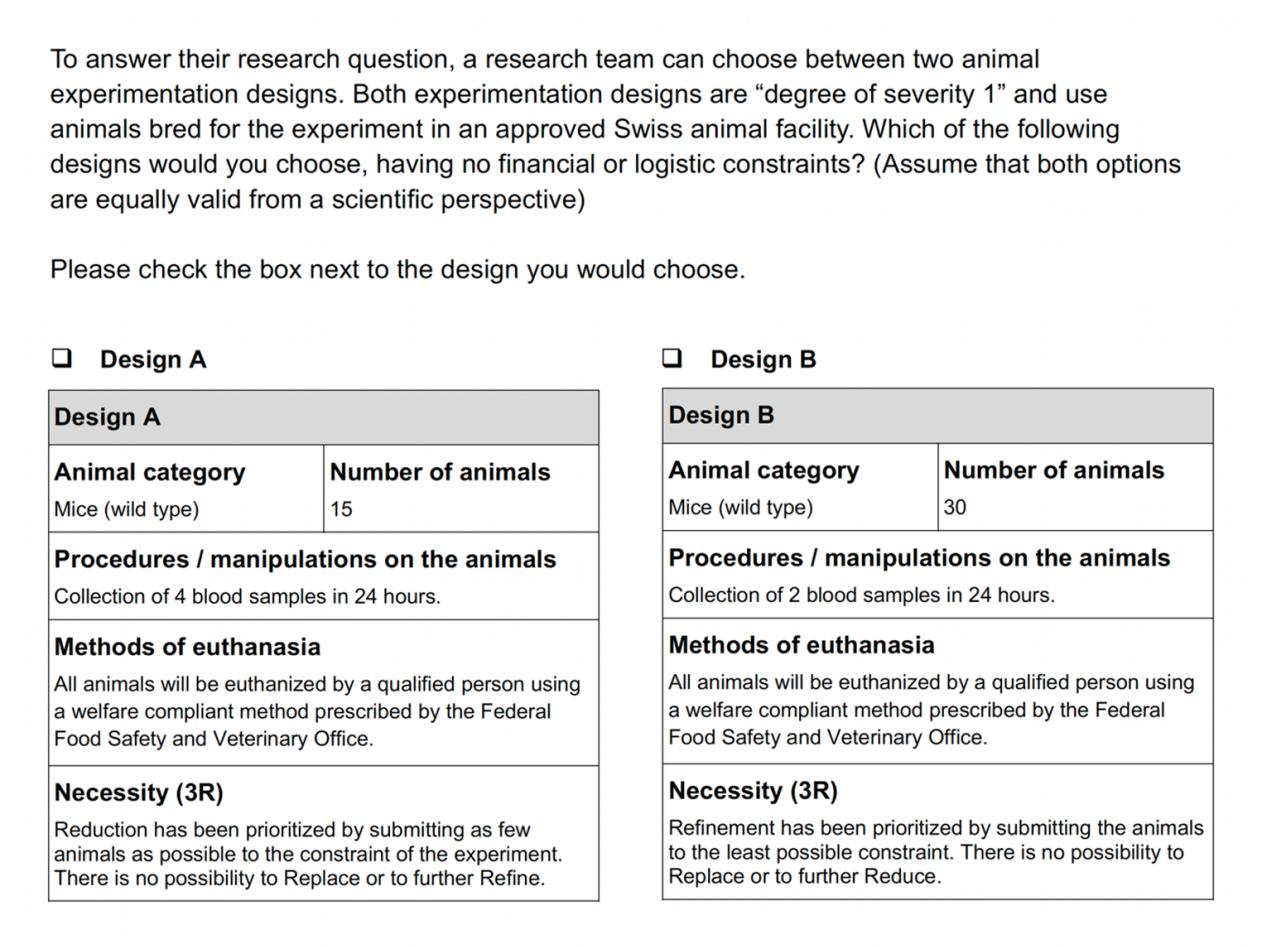
BioXPhi template for 3R dilemmas – Swiss APAE: Burden sharing – Probing fate: Euthanasia CVT

**Fig. 6:**
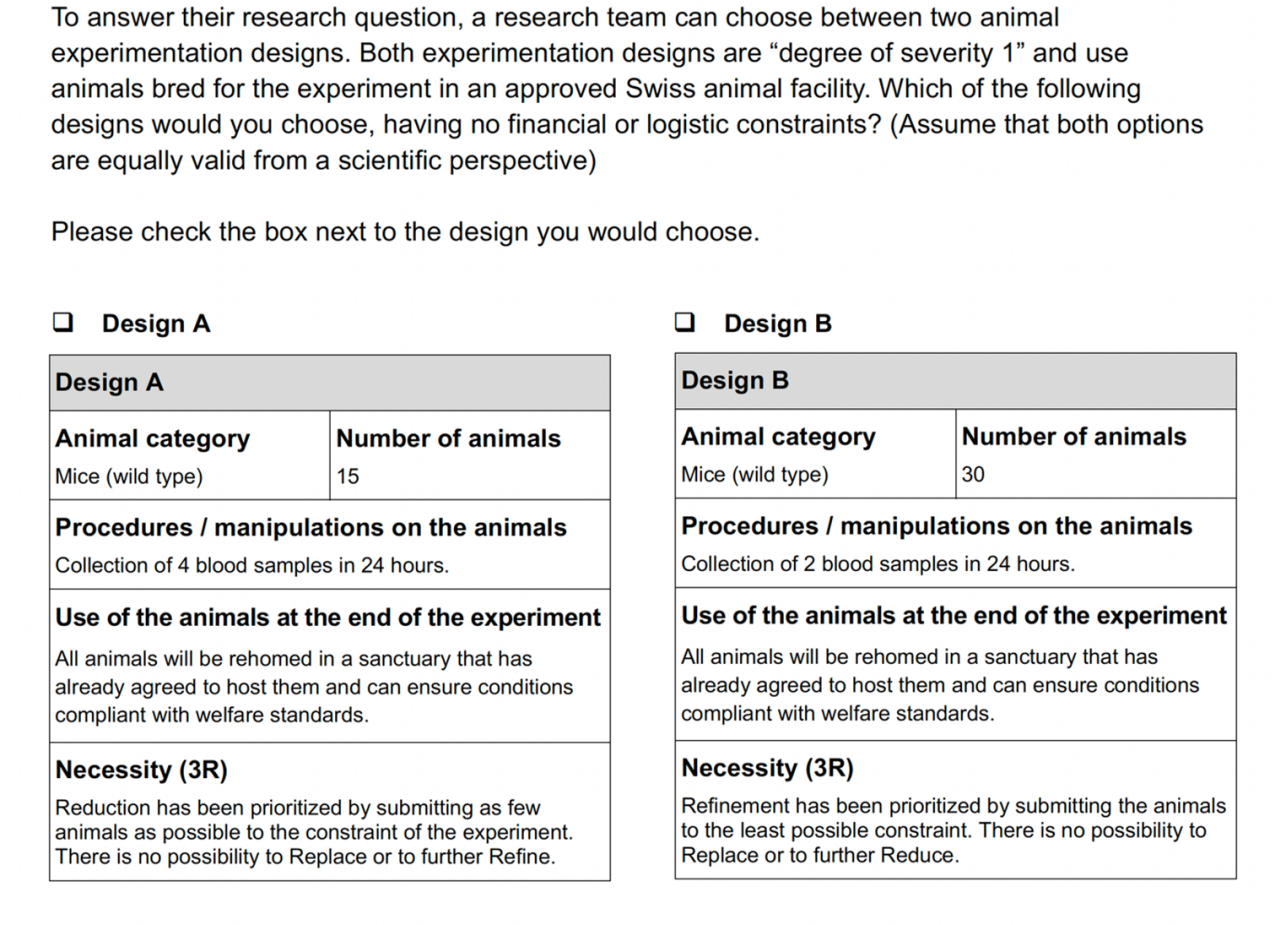
BioXPhi template for 3R dilemmas: – Swiss APAE: Burden sharing – Probing fate: Rehoming CVT

**Fig. 7:**
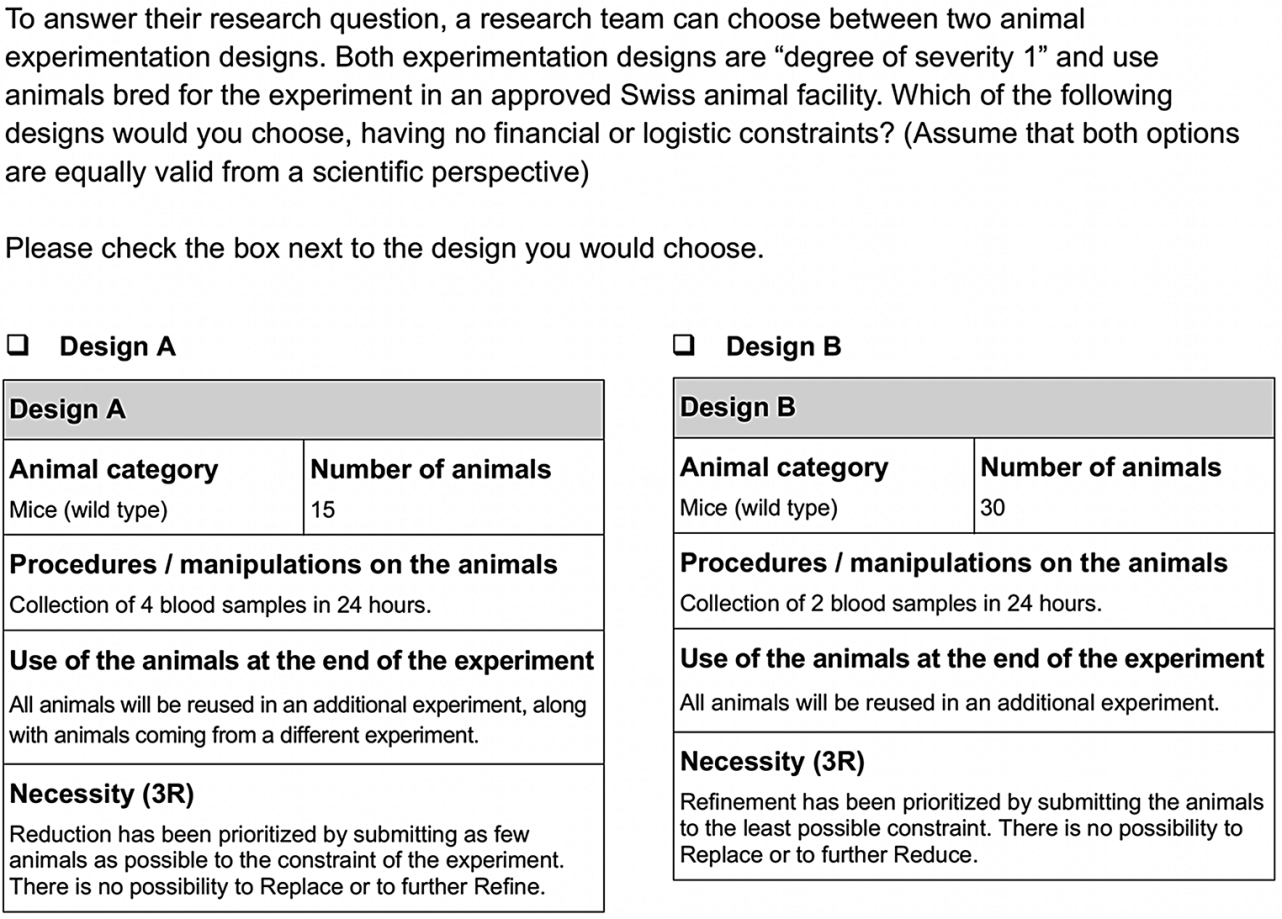
BioXPhi template for 3R dilemmas: – Swiss APAE: Burden sharing – Probing fate: Reuse CVT

Below, we present a bioxphi template for 3R dilemmas. We show a three-case template (Figs. 5, 6 and 7) which enables experimental probing of the fate factor in a burden sharing 3R dilemma. In this case, our template complies with the Swiss APAE in its content, wording, and design, to favor ecological validity. This approach must be followed when applying the template to other contexts, based on the APAE of reference. We will then conclude by giving recommendations on the use of the template in experiments, exploring the normative relevance of data collected by such means, and discussing important limitations.

### Recommendations and normative relevance

As we said, our three-cases (i.e., euthanasia, rehoming, reuse) template can be used to probe the fate factor in burden-sharing refinement-reduction dilemmas. And in doing so, it balances in each case the rationale for prioritizing reduction or refinement, in the “Necessity (3R)” section. The degree of severity 1 experienced by all animals has been defined according to the Swiss Federal Food Safety and Veterinary Office’s guidelines. The degree of severity does not change in the case of reuse in an additional experiment, which is consistent with the APAE’s process (i.e., the additional experiment requires a different APAE). To ensure that participants do not consider rehoming or reuse as not feasible, or as illegal, we specify that all mice are “wild type” (i.e., not genetically modified) and we use a standard case of blood sampling as the procedure. In the introduction of the case, all animals are presented as “bred for the experiment”, to avoid the possibility that participants prioritize refinement on the basis that a life in a degree of severity 1 experiment may be better for the animals than a life in another (more severe) experiment. Additionally, we emphasize that both designs are equally valid from a scientific perspective.

The template can be used with a digital survey tool allowing participants to select in each case their decision. To probe a particular factor, two different approaches can be taken. The first approach consists in submitting all three cases in random order to the same participants (i.e., “within-subjects” design). This approach would allow more data to be gathered within a fixed sample size, as well as to investigate how the order of the cases presented affects the prioritization of one of the Rs. The second approach consists in submitting only one case per participant (i.e., “between-subjects” design). This approach would require a bigger sample size to attain enough statistical power, but it would avoid “order effects” (Petrinovich and O’Neill 1996) or potential effects due to “experimenter demand” (Zizzo 2010). Both approaches will inform us on *why* and *how* participants made particular decisions, according to the probed factor. Additionally, we would recommend giving participants the opportunity to express their rationale for each decision (e.g., by using open-ended questions in the digital survey tool). This would ensure that as many considerations as possible are captured, potentially even those more difficult to anticipate (e.g., technical, economic, legal, or logistical ones).

Regarding participants, we said that a variety of professional decision-makers using the APAE could participate in such investigations and reveal key findings. In addition to researchers, ecological validity would also be strong with members of ARECs with a technical background, who are responsible for evaluating APAEs. Therefore, it would be interesting to look at differences between these groups of decision-makers, as well as differences within a group based on other variables (e.g., country, type of APAE, years of experience). Other decision-makers (e.g., members of authorities, veterinarian, statisticians, animal welfare specialists) might reveal interesting results as well, even if ecological validity would be weaker in the sense that they might not use the APAE directly for their decision-making.

Depending on the context (e.g., country’s legislations, current debates), some factors would be more urgent to probe. In our example we focused on the fate factor because many aspects of the Swiss and European context call for such investigation. The phenomena of compassion fatigue and moral distress among animal research decision-makers are often linked to the killing of animals. Moreover, cases of rehoming of healthy research animals at the end of experiments are still rare exceptions, and their reuse in additional experiment also creates diverging moral intuitions. But while there is a growing concern among the public and professionals when it comes to unnecessarily killing animals, we said that Swiss law considers the killing of animals in research as a degree of severity 0. It is to improve the situation of both animals and professionals in such complex issues that gathering data on these dilemmas is necessary.

How, then, could such data improve these situations? As we said, empirical research involving stakeholders can be useful for a variety of purposes. On the one hand, results can be used descriptively to identify moral issues that have escaped our attention or to learn more about decision-makers’ reasoning patterns, as well as about their understanding and awareness of regulations, guidelines, and definitions. On the other hand, they can be used to inform ethical or policy decisions whereby normative conclusions are drawn from argumentative premises that include empirical claims about the moral judgments of relevant stakeholders.

Bioxphi presents four approaches to making such normative inferences: parsimony (i.e., give *prima facie* normative weight to stakeholders’ consensus judgments if they survive appropriate tests of validity, robustness, and reliability across trials, measures, and manipulations), debunking (i.e., exclude or discount judgments resulting from unreliable decision-making processes, e.g., processes shown to be highly susceptible to inappropriately biasing factors or morally irrelevant framing effects; see Demaree-Cotton 2016), triangulation (i.e., seek coherence between stakeholders’ conflicting views, through a process of reflective equilibrium) and pluralism (i.e., give comparable normative weight to conflicting views, insofar as both/all prove similarly robust and/or resistant to debunking attempts) (Earp et al. 2021). We would argue that all four approaches are useful to make recommendations on how to deal with 3R dilemmas. Below, we give two concrete examples of how this could be done. First, we show how the parsimony approach could be used, by going back to the tension in the Swiss context between valuing research animals’ lives and dealing with a law considering their death as almost ethically irrelevant. Second, we discuss how a debunking approach could be used to address cases of speciesism.

- *The parsimony approach and the harm of death in animal research*.

First, imagine that after probing a variety of factors in different refinement-reduction dilemmas, the data consistently show that the fate factor has no significant influence on the prioritization of refinement or reduction by Swiss researchers. Moreover, imagine that most participants prioritize refinement over reduction, in line with the results of Franco and colleagues (Franco and Olsson 2014, Franco et al. 2018). In other words, whether the animals will be euthanized, rehomed, or reused at the end of the experiment does not significantly affect the decision regarding prioritization of refinement (i.e., inclusion of more animals).

On the one hand, such results would be consistent with the underlying normative assumption of current Swiss law, which does not consider the fate of the animal as ethically relevant. On the other hand, if participants in the rehoming conditions do not significantly differ in their decisions (compared to those in the euthanasia conditions), one could question the relevance of rehoming initiatives. Now imagine that participants used open-ended questions to express their rationale, giving reasons such as “[…] *I see that the animals will be rehomed*,* that is nice*,* but maybe euthanasia would have been better for laboratory animals who are not used to living outside the laboratory*”. Such qualitative additions could help us make sense of the results. Since the parsimony approach requires us to give *prima facie* normative weight to the most consistent and robust ethical judgments of relevant stakeholders (as described above), the results could be used to argue that current Swiss law does not need to revise its approach to the killing of research animals, at least insofar as the law aims to cohere with the (*prima facie*^5^ normatively weighty) moral attitudes of relevant stakeholders. Moreover, the results could be used to call for clear guidelines on how to judge in which specific cases rehoming is preferable to euthanasia, in turn ensuring that rehoming initiatives apply said guidelines.

Now let us consider the opposite scenario: imagine that after probing a variety of factors in different refinement-reduction dilemmas, the data consistently show that refinement is prioritized to a (significantly) lesser extent when the fate of the animals is euthanasia, compared to when their fate is rehoming or reuse. As an example, imagine that in rehoming and reuse conditions, most participants prioritize refinement over reduction, while in euthanasia conditions, decisions are approximately equally distributed between refinement and reduction. In other words, if the animals are going to be euthanized at the end of the experiment, participants are less willing to include more of them.

Such a result would still need interpretation, and qualitative inputs (along with follow-up studies to manipulate further potentially relevant factors identified through qualitative analysis) might again be useful. Let us imagine that among participants who prioritized reduction, their answers to open-ended questions express reasons such as “*If the additional animals are not reused this seems like a waste of resources*”, *“I prefer not to burden professionals with the task of euthanizing more animals”* or, in a case where thousands of animals are involved, “*In this case an excessive number of animals would have to die to comply with refinement*”. Such results would not only support the idea that giving animals “a life after the experiment” (i.e., rehoming) or optimizing their sacrifice (i.e., reuse) is significantly valued, they would also suggest that there is something deeply problematic about the way current Swiss law considers the killing of research animals.

Indeed, such results would show that key decision-makers base their 3R dilemma decisions on values that diverge from the law. While such results would not be too surprising, given the tension presented above, they would nonetheless call for a resolution of the issue. In this sense, the parsimony approach (perhaps in combination with triangulation) could be used to argue for a revision of the legal status of the killing of research animals, for example by increasing its degree of severity or, in line with Swiss law, by including it as a non-pathocentric harm to animal dignity (AniWA, art. 3a). These revisions would enable professionals to rely on the law when making 3R dilemmas decisions, or when planning initiatives to tackle compassion fatigue and moral distress. Giving *prima facie* normative weight to such consensus judgments would also enable the argument that support and incentives for rehoming initiatives are needed. While such concrete steps should be made by, or at least in collaboration with, the relevant experts and authorities (e.g., Federal Food Safety and Veterinary Office), we can see how bioxphi results can inform normative debate and policymaking. That is why we hope to contribute to these important issues with our upcoming study using this approach, and further hope that it will be used by others and adapted to different contexts.

b) *The debunking approach and speciesism*.

As the work of Franco and Olsson (2014) discussed above reveals, when (instead of mice) it comes to species like primates or dogs, researchers are less willing to use more animals, even if that implies inflicting more harm on individual animals. Our bioxphi template enables further investigation of *why* and *how* the species factor influences decision-making in such dilemmas, by systematically changing the species while controlling for other factors. According to some positions in animal ethics, the species of an animal can be relevant in moral decision-making, mainly due to the capacities attributed to the species. For example, when considering the harm of death for a particular species, the connectedness between the animal’s current and future self (i.e., the capacity to make plans or have desires for their future lives) can be considered relevant (McMahan 2016). However, when such distinctions are *merely* based on species (i.e., when they do not reflect morally relevant species-specific capacities), they are considered by most animal ethicists as cases of speciesism.

Speciesism can be defined as the assignment of different inherent moral status based solely on an individual’s species membership (Caviola et al. 2019). Because it solely depends on, or is too strongly influenced by, a morally irrelevant factor (i.e., mere species), speciesism is an unreliable process of moral decision-making, which is why the debunking approach is relevant here. As we said, the debunking approach excludes or discounts judgments resulting from such unreliable decision-making processes. Research in moral psychology has given us fascinating insights regarding humans’ speciesist attitudes (Caviola et al. 2019; Wilks et al. 2021) and there is no reason to exclude the possibility that these also play a role in 3R dilemma decision-making. For example, suppose that manipulating species type leads to substantially different resolutions to a given dilemma, where, theoretically, species type alone should make no difference from a moral perspective. And then a follow-up study might probe these judgments further and show that they are due to an irrational prejudice against the welfare of certain kinds of animals (e.g., perhaps ones that are not as “cute” as other ones, despite having similar needs). Based on the debunking approach, this would suggest that we should exclude or discount stakeholder judgments about certain animals (or enact an intervention to counteract the pro-cuteness bias) when designing the relevant policy.

### Limitations

While the proposed template has the advantage of favoring ecological validity for the group under study by complying with the APAE’s content, wording, and design, many situational aspects are not captured by it. We have seen that the completion of an APAE is the result of a process which takes time, and involves deliberation with peers, literature review as well as financial and deadline considerations, real-life aspects which are not present here. In the case of ARECs members and the evaluation of APAEs, similar real-life aspects and additional ones (e.g., scientists dominating discussions) are also missing (Azilagbetor et al. 2024). These aspects are important since decision-making in these contexts has been called into question for its biases and lack of transparency, contributing to calls for a paradigm shift (Herrmann 2019). While an extensive discussion of these issues falls outside the scope of this paper, we must keep in mind that the template does not recreate real-life situations nor provide results corresponding exactly to real-life decisions participants would make. Nonetheless, as we have shown in the example above, the template enables the investigation of the *whys* and *hows* of 3R dilemma decision-making in a way that provides important results.

It could be argued that APAEs are disconnected from researchers’ 3R dilemma decision-making process, meaning that they are merely filled once the decision is already made. But such a claim lacks plausibility for several reasons. First, researchers with some experience with the APAE should already have in mind the process by which 3R decisions must be explained and justified. Second, in the case of unexperienced researchers, we can imagine that being confronted with the APAE could even lead to a revision of the initial decisions. Finally, since completion, submission, and validation of the APAE is such a decisive, long, and demanding process, instead of two distinct phases (i.e., decision and completion) it rather seems to imply an overlap in which decisions and completion are made simultaneously. In any case, even if it were true that APAEs are disconnected from researchers’ 3R dilemma decision-making process (which as we just argued is very unlikely), the template would still be useful for other decision-makers, such as members of ARECs with a technical background.

## Conclusion

In this article, we argued for the necessity to investigate 3R dilemmas in animal research using experimental bioethics (“bioxphi”). In a first step, we presented what 3R dilemmas are and discussed previous investigations of professionals’ attitudes in such cases. In a second step, we showed that bioxphi is ideally tailored to go beyond *what* decision-makers decide in 3R dilemmas and reveal *why* and *how* they do so. There, we presented the applications to perform an animal experiment (APAE), and key factors which can be found in them, as crucial tools to favor ecological validity in such investigation. In a last step, we presented a bioxphi template for 3R dilemmas, gave recommendations on its use, explored the potential normative relevance of data collected by such means, and discussed important limitations. The template complied with the Swiss APAE in its content, wording, and design to favor ecological validity, an approach that we suggest should be followed when applying the template to other contexts.

As we have shown, the APAE is an essential part of animal researchers’ work, but also of other decision-makers such as members of animal research ethics committee. The decisions they must make about the suffering, and often the death, of vast numbers of non-human animals have important ethical consequences. This is true not only for research animals, but also for professionals themselves who are deeply affected by these decisions, as phenomena such as compassion fatigue and moral distress attest. As we argued, knowing more about the factors and processes shaping decision-making in these dilemmas can help inform both theoretical and policy-oriented discussions around these issues. To exemplify this, we discussed the consideration of death as a harm in the context of Switzerland, as well as cases of speciesism in 3R decision-making. But the contribution of bioxphi to the field of empirical animal ethics does not end with 3R dilemmas or the APAE. Other tools used daily by professionals working with animals in different fields represent an opportunity to ensure context-specificity and ecological validity while experimentally testing attitudes and behaviors on important ethical issues. Bioxphi must indeed become an addition to the toolbox of empirical animal ethicists, and investigating 3R dilemmas will be an important first step.

## Data Availability

Not applicable.

## Footnotes

1 Switzerland’s APAE can be accessed in the reference list: Swiss Confederation. Federal Food Safety and Veterinary Office. Application for licence to perform animal experiment.

2 United Kingdom’s APAE can be accessed in the reference list: UK Government. Project licence application under the Animals (Scientific Procedures) Act 1986.

3 The European Union requires Member States to establish a national committee which shall evaluate projects (see Directive 2010/63/EU, Art. 49, 2, in the reference list). Canadian provinces have different legislations on animal welfare which regulate the issuance of licences (see the Canadian Council on Animal Care (CCAC) in the reference list). In the United States, an example of institutional protocol review is conducted by the Institutional Animal Care and Use Committee (see National Institutes of Health in the reference list).

4 The Swiss Federal Food Safety and Veterinary Office (FSVO) defines four degrees of severity in the context of animal research, which include severity 0–3 where: 0 equals no harm; 1 equals mild harm; 2 equals moderate harm and 3 equals severe harm.

5 The qualification ‘prima facie’ is important. It simply suggests that the most consistent (i.e., widely-shared and internally coherent), robust and reliable moral judgments of relevant stakeholders – as identified through controlled bioxphi experiments, which may include (failed) attempts to empirically *debunk* such judgments – should be given *some* nonzero normative weight in arguments about what the right (or morally best justified) policy or course of action would be. This is different from saying that such judgments should automatically (or all-things-considered) determine policy decisions; after all, they might be outweighed by other important considerations, be they practical or moral. See Earp et al. (2021) for an extended discussion.
